# Do the selection criteria of internal medicine residency program predict resident performance?

**DOI:** 10.5339/qmj.2021.20

**Published:** 2021-06-21

**Authors:** Ali Rahil, Tahseen Hamamyh, Ahmed Al-Mohammed, Antoun Kamel, Ibrahim Abubeker, Laith Abu-Raddad, Soha Dargham, Shireen Suliman, Dabia Al Mohanadi, Abdulatif Al Khal

**Affiliations:** ^1^Hamad General Hospital, Doha, Qatar E-mail: arahil@hamad.qa; ^2^Biomathematics Research Core, Weill Cornell Medical College, Qatar

**Keywords:** USMLE, ITE, medical education, ACGME-I

## Abstract

Background: Well-performing physician reflects the success of the residency program in selecting the best candidates for training. This study aimed to evaluate the selection criteria, mainly the United States Medical Licensing Examination (USMLE) Step 2 Clinical Knowledge (CK) results and applicants’ status as international or locally trained applicants, used by the medical education department and the internal medicine residency program in Hamad Medical Corporation in Qatar to predict the residents’ performance during their training.

Methods: A retrospective chart review was performed for three batches of graduates who started residency training in 2011, 2012, and 2013. Each group completed 4 years of training. The USMLE Step 2 CK status of the applicant, in-training exam (ITE) scores, formative evaluation scores, Arab Board written and clinical exams pass rate, and other indicators were analyzed. Statistical analysis included chi squares and independent t-test to identify associations. Multivariable analyses were conducted using logistic and linear regressions to test for adjusted associations.

Results: The study included 118 (81 international/37 locally trained applicants) internal medicine residents. The ITE score correlated positively with the USMLE Step 2 CK score (r = 0.621, r = 0.587, r = 0.576, r = 0.571, *p* < 0.001) over the 4 years of training and among the international compared with locally trained applicants (*p* < 0.001). The rate of passing part 1 and 2 written exam of the Arab Board was higher in international than in local applicants, whereas clinical Arab Board exam and formative evaluation were not associated with any criteria.

Conclusions: Higher USMLE Step 2 CK score correlated with better performance on ITE but not with other performance indicators, whereas international applicants did better in both ITE and Arab Board written exam than local applicants. These variables may provide reasonable predictors of well-performing physicians.

## Introduction

Selecting medical graduates for a residency training program is confronted with a challenge of identifying the best applicants who will perform well both as residents and later as future independent physicians. Although no universally agreed upon selection criteria can predict ideal candidates, most programs depend on standardized examination scores such as the United States Medical Licensing Examination (USMLE) Step 1 and Step 2 Clinical Knowledge (CK), in addition to personal interviews to decide whether an applicant can be offered a resident position.^1^

USMLE is part of the official requirement to gain medical licensure to practice medicine in the United States.^2^ Its score was not originally intended to be used in the residency acceptance process. However, it is considered by many program directors as an important factor in prioritizing applicants, even though studies have shown conflicting results on the reliability of USMLE scores in predicting a resident's performance.^3-5^

Resident performance has been assessed differently in various studies. Some studies have concentrated on clinical and communication skills,^6,7^ whereas others have focused on in-training exam (ITE) results or passing the American board exam of a specific specialty.^8-10^ The Accreditation Council for Graduate Medical Education (ACGME) defined the milestones of residents’ evaluation to measure their performance in six competencies: medical knowledge, patient care, systems-based practice, practice-based learning and improvement, professionalism, and interpersonal and communication skills.^11^ Brittany *et al.* demonstrated a positive correlation between structured interviews, USMLE Step 1 and 2 results, and residents’ performance in ACGME competencies in 14 US residency programs.^12^ The internal medicine residency program at Hamad Medical Corporation (HMC) in Qatar is an ACGME-International (ACGME-I)-accredited academic program since 2013. Fifty medical graduates from different universities, ethnicities, and national backgrounds are matched annually in our program, which are selected from more than 300 annual applicants. Taking into account the wide diversity in candidates’ medical background and the differences in their academic transcript, the resident selection criteria depend largely on standardized examination scores such as the USMLE Step 2 CK, besides giving priority to graduates who completed the internship training at HMC.

However, whether the USMLE-CK or any other criterion correlates well with the resident's performance in the internal medicine residency program in our institution or any other ACGME-I-accredited institutions has never been explored.

In this retrospective study, we aimed to explore the reliability of the selection criteria, mainly USMLE Step 2, used by the internal medicine and medical education departments at HMC in predicting the performance of residents.

## Methods

### Study design and data analysis

This study is a retrospective chart review of electronic and paper-based internal medicine residents’ files from 3 intake years (2011, 2012, and 2013) each consists of 4 years of training in HMC, Qatar. We tested the association of the selection criteria including USMLE Step 2 CK, being local applicants (as opposed to international) with a residency permit, or undertaking the internship in HMC with the residents’ performance measured by score on formative evaluations, passing Arab Board Part 1 and Part 2 written exams from the first attempt, and the ITE scores.

Sample characteristics were summarized using frequency distributions for categorical variables and mean and standard deviation for continuous variables. Chi-squares and independent t-tests were used as appropriate to identify crude associations. Multivariable analyses were conducted using logistic and linear regressions to test for adjusted associations. Crude and adjusted odds ratios (OR) and beta (ß) coefficients were reported along with the respective 95% confidence intervals (CI). Significance was identified at the 5% level. All analyses were conducted using IBM-SPSS 24.0 (IBM Corp., Armonk, NY, USA).

### Study populations

A total of 118 locally trained (who had a residency permit or completed their internship in Qatar) and international applicants joined the internal medicine residency program between 2011 and 2013. Moreover, 88 (75%) residents completed the training period, whereas the remaining 30 (25%) residents left the program at different levels of training (7 candidates left the program in the second year, 10 in the third year, and 13 in the fourth year of the residency training) to join the residency programs in the United States. The program is a hybrid of an ACGME-I accredited program and an Arab Board program. The training period spanned for 4 years: 3 years to complete the ACGME curriculum and another 1 year as required by the Arab Board program with an exit exam for Arab Board certification.

### Selection criteria

The medical education department of HMC (main training institution) mandates certain requirements for applicants for them to be shortlisted for interview with the internal medicine program director. These include medical school certificate, English language proficiency test (which is the International English Language Testing System with a minimum score of 7 or Test of English as a Foreign Language), USMLE Step 2, evidence of the lack of a career gap, in addition to any other extracurricular activities and scholarly activity. Of these criteria, USMLE has been considered the most important criterion to select candidates for the program based on a score of >230 (out of 300) for international applicants and >208 (out of 300) for locally trained applicants.

### Performance indicators

Residents’ performance during their training program was assessed across different domains/outcomes that included (i) ITE, which is a standardized multiple-choice question exam validated by the American Board of Internal Medicine (ABIM) score of each residency year; (ii) the cumulative score of the formative evaluation, which was adapted from that of ACGME and performed by the teaching faculty at the end of each rotation; the score is based on rating from 1 to 5 (where 1 is unsatisfactory and 5 is outstanding) and included the six competency domains of the ACGME; (iii) Arab Board (part 1 and 2) written exam result as pass or fail from the first attempt; and (iv) Arab Board Clinical Exam result as pass or fail from the first attempt.

### Ethical approval

The study was considered as service evaluation by the HMC Institutional Review Board (IRB) and exempted from IRB approval (MRC-01-20-887). All analyses were conducted on de-identified database.

## Results

A total of 118 applicants were included in the study; 94 (79%) were men and 24 (21%) were women. From these applicants, 81 (69%) were international applicants, whereas 37 (31%) were locally trained applicants (Table 1).

(a) ITE (Table 2)

   ITE scores over the 4 years of residency showed a positive correlation with USMLE Step 2 score and other variables.

   Higher ITE scores were seen in international applicants than in those who had a residency permit status in Qatar (local in Qatar) and those who had their internship completed in Qatar.

   The ITE score was higher in male graduates than in female graduates with a mean value ranging between 59% and 76% and between 55% and 69%, respectively, across the 4 years of training. This difference was significant for postgraduate year (PGY)2 (*p* = 0.008), PGY3 (*p* = 0.006), and PGY4 (*p* = 0.004). However, after adjustment for confounders, only the USMLE score remained significant at a beta (β) coefficient that ranged between 0.06 and 0.10 across the 4 years of ITE. The median USMLE score for local applicants was 215 compared with 234 for international applicants (*p* < 0.001).

(b) Formative Evaluation (Table 3)

   None of the variables, including USMLE score, were associated with the residents’ evaluation by the teaching faculty. However, the formative evaluation showed marginally significant association with local applicants (*p* = 0.051)

(c) Arab Board Written Exam (Tables 4 and 5)

   A negative correlation was shown between being a local applicant at the time of application and passing the Arab Board part 1 (OR 0.16; 95% CI 0.03–0.89; *p* = 0.037) and part 2 (OR 0.09; 95% CI 0.01–0.80; *p* = 0.037) written exams. Moreover, having an internship in Qatar was negatively correlated with passing the Arab Board 2 written exam (OR 0.13; 95% CI 0.02–0.75; *p* = 0.022). Male graduates were more likely to pass the second part of the exam (*p* = 0.046). However, none of these associations remained significant in the multivariable analyses.

(d) Arab Board Clinical Exam (Table 6)

   None of the variables, including the USMLE score, were significantly associated with the passing of Arab Board Clinical Exam. However, results of Arab Board part 1 were borderline significant in predicting the result of the Arab Board Clinical Exam (*p* = 0.086). Residents who passed the Arab Board part 1 were six times (OR 5.6; 95% CI 0.8–37.6) more likely to pass the Arab Board Clinical Exam than do their counterparts (OR 5.6; 95% CI 0.8–37.6).

## Discussion

Our study demonstrated that the main selection criteria used by our program, that is, USMLE Step 2 score, had a significant association with the ITE results across the 4 years of residency even after adjustment for confounders. This association appears logical given the nature of both USMLE-CK and ITE as a knowledge exam. ITE is intended to assess medical knowledge and identify gaps in knowledge and has been shown to predict the performance on the ABIM Certifying Exam,^13-19^ the passing rate of which is used by ACGME to evaluate the quality of a residency training program. Although ITE is designed for PGY2 level, our study showed no difference between the different PGY levels, and the USMLE score and correlation was positively maintained throughout the 4 years of residency. Jose *et al.* concluded that USMLE Step 2 had the highest correlation with ITE than with USMLE Steps 1 and 3.^20^

In a meta-analysis, Kenny *et al* (2013) included more than 40,000 participants, and USMLE Step 1 was found to have the strongest association with the examination-based outcomes, such as ITE scores over different subspecialty programs, not only internal medicine, whereas USMLE Step 2 showed weaker association.^21^ Studies performed in orthopedic residency programs have also demonstrated a correlation between USMLE Step 1 and Step 2 and the orthopedic ITE.^22^ By contrast, our study showed that USMLE Step 2 was not a good predictor of the resident performance as captured by the composite score of the formative evaluation in all six ACGME competency domains, although there was a tendency for positive correlation among local applicants compared with international applicants (*p* = 0.051), which is not fully explained because international applicants have higher USMLE scores than local applicants. Thus, it may be attributed either to chance or to the subjective nature of the evaluation by the faculty. Not enough data are available to assess such association in internal medicine programs. However, USMLE Step 2 showed a positive association with the evaluation performance by program directors of surgical programs.^23^ Moreover, our study showed no correlation between USMLE score and other variables, particularly the Arab Board written exam (parts 1 and 2) despite being a knowledge exam. This finding may be related to the lack of score gradient similar to ITE and is based on the pass/fail criteria or possibly because of the small sample size where an effect may not have reached significance. The Arab Board Clinical Exam, which is an exit exam for the Arab Board and tests the clinical skills of history taking and physical examination in addition to clinical judgment, did not correlate with any of the criteria studied including the USMLE-CK. This finding needs to be taken cautiously because the exam is not well standardized and lacks validity evidence.

Depending on USMLE scores to predict the performance of residents in terms of clinical judgment, procedural skills, communication and other non-cognitive skills is not well supported by evidence. However, Norcini *et al.* showed a significant inverse relationship between USMLE Step 2 CK and in-hospital mortality from myocardial infarction or heart failure, emphasizing that the content of USMLE Step 2 CK may provide the foundation for safe and competent practice of medicine by graduating residents in such programs.^24^ In another study, Cuddy *et al.* found a positive correlation between higher USMLE Step 2 CK scores and lower odds of having a disciplinary action from a US state medical board.^25^ These observations highlight the fact that USMLE-CK might be a reasonable test to predict safe and competent practice.

Our study also revealed that international applicants did better than local ones in terms of the Arab Board written exam pass rate and ITE exam scores.

### Strength of the study

To the best of our knowledge, this is the first study conducted in an ACGME-I accredited program. The study included only an internal medicine residency program with a reasonable number of candidates enrolled. Our program is unique in that it is a mix of ACGME-I program and Arab Board program with a total of 4 years of training. Moreover, the study population was composed of graduates from diverse international schools and national origins.

### Study limitations

Our results might be subject to attrition bias because 25% of the residents left the program during the residency period for further training in the USA.

Selection bias might have been introduced while shortlisting local and international applicants, because local applicants with lower USMLE Step 2 score were more likely to be accepted than international applicants.

The cut-off score for the USMLE of 230 is arbitrary and was selected by the program to indicate high scores versus low scores.

The Arab Board written (both parts) and clinical exams have dichotomous result: either pass or fail, lacking the score gradient. The performance of residents as a predictor of future competent physician is not only dependent on medical knowledge but also on the composite of the six competency domains.

## Conclusion

To our knowledge, this study is the first in our institution and region to investigate the process of selecting residents for training program and highlighting the limitation of our selection criteria in recruiting internal medicine residents and thereby predicting the performance of residents during the training program that will ultimately shape the capacity and performance of future physician. In conclusion, the USMLE Step 2 score is probably not a robust tool in most of the outcome measures except for the cognitive knowledge tested by the ITE. International applicants showed better performance in cognitive tests than locally trained applicants. Further study is needed to determine the best combination of criteria that will predict competent internal medicine residents and future practicing physicians.

### Acknowledgement

The authors are grateful for the support provided by the Biomedical Research Program and the Biostatistics, Epidemiology, and Biomathematics Research Core, both at Weill Cornell Medicine-Qatar. The statements made herein are solely the responsibility of the authors.

### Conflict of interest

The authors do not have competing interest in relation to this paper.

### Funding

None

## Figures and Tables

**Table 1:** Demographic and baseline characteristics

Variables	Number (%)/Mean (SD)

Total number of candidates	118
Year 2011	34 (29%)
Year 2012	42 (36%)
Year 2013	42 (36%)
Gender	
Male	94 (80%)
Female	24 (20%)
Qatar immigration residency (local training)	
Yes	37 (31%)
No	81 (69%)
Internship in Qatar	
Yes	25 (21%)
No	93 (79%)
Mean USMLE score (SD)	229.6 (18.4)
Number completed the program	
Yes	88 (75%)
No	30 (25%)

**Table 2:** Crude association of ITE scores for years 1–4 with the variables

Variable	ITE-Year 1 Score	ITE-Year 2 Score	ITE-Year 3 Score	ITE-Year 4 Score

	N; mean score (SD)	N; mean score (SD)	N; mean score (SD)	N; mean score (SD)
	*p* value	*p* value	*p* value	*p* value
USMLE score				
< 230	34; 0.53 (0.09)	54; 0.60 (0.08)	53; 0.66 (0.08)	51; 0.71 (0.08)
> = 230	37; 0.63 (0.07)	53; 0.63 (0.09)	43; 0.75 (0.09)	34; 0.80 (0.08)
	(*p* < 0.001)	(*p* < 0.001)	(*p* < 0.001)	(*p* < 0.001)
USMLE (linear)	N = 71; r = 0.621	N = 107; r = 0.587	N = 96; r = 0.576	N = 85; r = 0.571
	(*p* < 0.001)	(*p* < 0.001)	(*p* < 0.001)	(*p* < 0.001)
Gender				
Male	56; 0.59 (0.10)	89; 0.64 (0.10)	79; 0.70 (0.11)	69; 0.76 (0.10)
Female	17; 0.55 (0.08)	21; 0.58 (0.07)	20; 0.65 (0.06)	18; 0.69 (0.06)
	(*p* = 0.200)	(*p* = 0.008)	(*p* = 0.006)	(*p* = 0.004)
Qatar immigration status (local)				
Yes	25; 0.52 (0.10)	35; 0.58 (0.11)	33; 0.65 (0.10)	31; 0.70 (0.09)
No	48; 0.61 (0.08)	75; 0.65 (0.09)	66; 0.72 (0.10)	56; 0.77 (0.09)
	(*p* < 0.001)	(*p* < 0.001)	(*p* < 0.001)	(*p* < 0.001)
Internship (local)				
Yes	18; 0.52 (0.11)	25; 0.59 (0.11)	23; 0.65 (0.09)	23; 0.70 (0.09)
No	55; 0.60 (0.08)	85; 0.64 (0.09)	76; 0.71 (0.10)	64; 0.76 (0.09)
	(*p* < 0.001)	(*p* = 0.008)	(*p* = 0.016)	(*p* = 0.003)

-SD, standard, deviation; r, Pearson's correlation coefficient

**Table 3:** Crude association of formative evaluation scores with the variables

Variable	N; Mean score (SD)	*p* value

USMLE score		
< 230	44; 4.16 (0.31)	0.349
> = 230	48; 4.10 (0.26)	
Gender		
Male	75; 4.10 (0.24)	0.117
Female	19; 4.24 (0.38)	
Qatar immigration status (local)		
Yes	29; 4.21 (0.32)	0.051
No	65; 4.09 (0.25)	
Internship (local)		
Yes	21; 4.21 (0.32)	0.119
No	73; 4.10 (0.27)	

-SD, standard deviation

**Table 4:** Crude association of Arab Board part 1 exam scores with the variables

Variable	Pass N (%)	Fail N (%)	OR (95% CI)	*p* value

Gender				
Male	85 (82.5)	4 (57.1)	3.54 (0.73–17.21)	0.117
Female	18 (17.5)	3 (42.9)	Ref	
USMLE score				
< 230	52 (51.0)	1 (20.0)	4.16 (0.45–38.51)	0.209
> = 230	50 (49.0)	4 (80.0)	Ref	
Qatar immigration status (local)				
Yes	30 (29.1)	5 (71.4)	0.16 (0.03–0.89)	0.037
No	73 (70.9)	2 (28.6)		
Internship (local)				
Yes	23 (22.3)	2 (28.6)	0.72 (0.13–3.95)	0.740
No	80 (77.7)	5 (71.4)		

-OR, odds ratio; CI, confidence interval

**Table 5:** Crude association of Arab Board part 2 exam scores and the variables

Variable	Pass N (%)	Fail N (%)	OR (95% CI)	*p* value

Gender				
Male	54 (79.4)	3 (42.9)	5.14 (1.03–25.68)	0.046
Female	14 (20.6)	4 (57.1)	Ref	
USMLE score				
< 230	28 (42.4)	2 (28.6)	1.84 (0.33–10.19)	0.484
> = 230	38 (57.6)	5 (71.4)	Ref	
Qatar immigration status (local)				
Yes	24 (35.3)	6 (85.7)	0.09 (0.01–0.80)	0.031
No	44 (64.7)	1 (14.3)		
Internship (local)				
Yes	17 (25.0)	5 (71.4)	0.13 (0.02–0.75)	0.022
No	51 (75.0)	2 (28.6)	Ref	

-OR, odds ratio; CI, confidence interval

**Table 6:** Crude association of Arab Board Clinical Exam results and the variables

Variable	Pass N (%)	Fail N (%)	OR (95% CI)	*p* value

Gender				
Male	35 (76.1)	11 (23.9)	1.06 (0.28–3.97)	>0.999
Female	12 (75.0)	4 (25.0)	Ref	
USMLE score				
< 230	26 (72.0)	10 (27.8)	1.92 (0.53–7.05)	0.319
> = 230	20 (83.3)	4 (16.7)	Ref	
Qatar immigration status (local)				
Yes	19 (70.4)	8 (29.6)	0.59 (0.18–1.91)	0.380
No	28 (80.0)	7 (20.0)	Ref	
Internship (local)				
Yes	15 (78.9)	4 (21.1)	1.29 (0.35–4.72)	>0.999
No	32 (74.4)	11 (25.6)	Ref	

-OR, odds ratio; CI, confidence interval
